# *Bacillus subtilis* ED24 Controls *Fusarium culmorum* in Wheat Through Bioactive Metabolite Secretion and Modulation of Rhizosphere Microbiome

**DOI:** 10.1007/s00248-025-02590-5

**Published:** 2025-08-19

**Authors:** Fatima Ezzahra Oulkhir, Abdelmounaaim Allaoui, Ayoub Idbella, Mohammed Danouche, Adnane Bargaz, Latefa Biskri, Mohamed Idbella

**Affiliations:** 1 AgroBioSciences (AgBS) Program College of Agriculture and Environmental Sciences, Mohammed VI Polytechnic University, Lot 660, Hay Moulay Rachid, Ben Guerir, 43150 Morocco; 2 Mohammed VI Polytechnic University, Lot 660, Hay Moulay Rachid, 43150 Ben Guerir, Morocco; 3 Chemical and Biochemical Sciences (CBS), Mohammed VI Polytechnic University, Lot 660, Hay Moulay Rachid, Ben Guerir, 43150 Morocco

**Keywords:** Endophyte, *Ziziphus lotus* (L.) Desf., Tebuconazole, *Triticum durum*, Rhizosphere microbiota, Secondary metabolites, Propionic acid

## Abstract

*Fusarium culmorum* is a soil-borne fungal pathogen causing root and stem rot, seedling blight, and significant yield losses in small grain cereals, including wheat. This study aimed to evaluate the antifungal potential of *Bacillus subtilis* ED24, an endophytic strain isolated from *Ziziphus lotus* (L.) roots, and its effects on wheat growth and yield under controlled conditions. In vitro assays demonstrated that *B. subtilis* ED24 inhibited *F. culmorum* mycelial growth by up to 87%, associated with the secretion of 37 distinct secondary metabolites, predominantly involved in carbon cycling. In pot experiments, *B. subtilis* ED24 significantly enhanced wheat germination (85%) and growth compared to infected plants treated with the chemical fungicide tebuconazole. Although nutrient analysis showed significantly higher shoot nitrogen (32.34 mg/pot) and phosphorus (2.41 mg/pot) contents in the *B. subtilis* ED24 treatment compared to tebuconazole (8.11 and 0.18 mg/pot, respectively), no significant differences were observed when compared to the infected control (C-). Similarly, *B. subtilis* ED24 led to improved thousand grain weight (40.4 g), protein content (19.98%), and ash content (1.95%) relative to tebuconazole (29.1 g, 18.31%, and 1.74%, respectively), yet these values did not differ significantly from the infected control (C-). Notably, the number of seeds per pot was significantly increased by *B. subtilis* ED24 compared to the infected control (C-) (113.8 seeds/pot vs. 54.2 seeds/pot). Additionally, *B. subtilis* ED24 modulated the wheat rhizosphere microbiome, enriching beneficial taxa such as *Eurotiomycetes* fungal class and the bacterial genus *Paramesorhizobium.* These findings suggest that the antifungal activity and growth-promoting effects of *B. subtilis* ED24 are likely mediated through the synthesis of unique bioactive metabolites and microbiome modulation, offering a promising sustainable alternative to chemical fungicides in wheat production.

**Supplementary Information:**

The online version contains supplementary material available at 10.1007/s00248-025-02590-5.

## Introduction

Fungal pathogens represent a major constraint to wheat production worldwide, causing yield losses of up to 40% [1]. Among them, *Fusarium culmorum* is the main fungal pathogen affecting cereal crops, particularly wheat, causing seedling blight, foot and root rot, and leading to significant losses in both grain yield and quality [2]. Chemical fungicides, notably azoles such as tebuconazole, have been widely applied to manage such pathogens. The chemical agents disrupt fungal membrane integrity by inhibiting sterol biosynthesis [3]. However, increasing concerns over environmental toxicity, human health risks, and the emergence of resistant fungal strains due to prolonged usage [4, 5] urgently call for environmentally friendly alternatives, such as biopesticides derived from microorganisms with antagonistic activity against targeted pathogens. Among them, the endophytic bacteria have emerged as key contributors due to their ability to reside inside plant tissues, enabling efficient exchanges of metabolites and signaling molecules [6]. Endophytes have co-evolved with their host plants, acquiring genetic traits that mimic or complement plant-derived compounds [7]. This co-evolution is particularly evident in medicinal plants known for their high content in bioactive compounds [8]. In this context, *Ziziphus lotus* (L.) Desf., commonly known as “Sedra” in Morocco, represents a promising source of novel endophytes. It is a medicinal plant with significant ethnobotanical value. Phytochemical investigations have identified around 431 chemical constituents [9], reflecting its extensive use in traditional medicine.

Considering the ecological and therapeutical features of *Z. lotus* (L.) Desf., the present study explores the microbial attributes of this medicinal plant by investigating the antifungal activity of *B. subtilis* ED24, an endophytic bacterium isolated from its roots. In addition to being characterized for its plant growth-promoting (PGP) properties, *B. subtilis* ED24 was evaluated in vitro for its biocontrol potential against the most prevailing phytopathogenic fungi, *Fusarium culmorum*, *Penicillium cyclopium*, and *Alternaria infectoria* with particular attention paid to secondary metabolite production. The second part of the study assesses the *in planta* biocontrol efficacy of the strain against *F. culmorum* under controlled conditions using durum wheat, variety Karim, known for its sensitivity to *F. culmorum*. This phase compares the effects of *B. subtilis* ED24 on seed germination, wheat growth, and yield to those of the commercial fungicide tebuconazole.

## Material and Methods

### Strains and Culture Conditions

This study involved four microorganisms. *B. subtilis* ED24, a root endophytic bacterium isolated from *Z. lotus* (L.) Desf., was identified by 16S rRNA gene sequencing (GenBank accession no. PQ620260), characterized, and utilized as a plant growth-promoting rhizobacterium (PGPR). The initial key PGP traits of *B. subtilis* ED24 were phosphate (108 mg/L) and potassium (7.71 mg/L) solubilization measured in liquid bacterial culture in NBRIP [10] and Aleksandrov [11] medium, respectively. *B. subtilis* ED24 was also able to grow on nitrogen-free Burk’s [12] medium, indicating nitrogen fixation ability. To test the anti-fungal activity of *B. subtilis* ED24, three pathogenic fungi, *Fusarium culmorum* (F8), *Penicillium cyclopium* (F10), and *Alternaria infectoria* (F19), were originally isolated from the wheat rhizosphere [13, 14]. *B. subtilis* ED24 was cultured at 30 °C in tryptic soy broth (TSB, Biokar Laboratories) for 24 h with shaking at 180 rpm. Using the Quantom™ XT Microbial Cell Counter (Logos Biosystems), an OD600 of 0.8 was determined to correspond to approximately 1 × 10^8^ CFU/mL. This value was used to standardize the bacterial suspension prior to inoculation. The fungal strains were grown on potato dextrose agar (PDA, Biokar Laboratories) and incubated at 28 °C for 7 days.

### Antifungal Activity Assay of B. subtilis ED24

*B. subtilis* ED24 was tested for its potential to act as a biocontrol agent against the three fungal pathogens mentioned above, i.e., *F. culmorum* (F8), *P. cyclopium* (F10), and *A. infectoria* (F19). These pathogenic strains were found to be the most prevalent in Morocco, affecting a significant portion of wheat culture [13, 15]. On PDA, a 5 mm disc from a 1-week-old sporulating mycelium of each fungal strain was placed at the edge of the plate. Approximately 10 µL of the *B. subtilis* ED24 bacterial culture was inoculated at the opposite edge of the plate. The plates were then incubated at 28 °C for 5 days along with control treatments consisting of the fungi and *B. subtilis* ED24 inoculated separately on individual plates. After incubation, the colony diameter of the fungi was measured. The percentage of fungal growth inhibition was calculated using the following formula [16]:

$$I=\frac{C-T}{C} \times 100$$

where *I* = inhibition percentage, *C* = radial growth of the pathogen in the control, and *T* = radial growth of the pathogen in the treatment.

### Determination of Antifungal Secondary Metabolites

#### Protease Production

*B. subtilis* ED24 was spotted onto nutrient agar medium supplemented with casein as a source of protein [17] and incubated at 30 °C for 48 h. The presence of halos indicated the production of extracellular protease.

#### Cellulase Production

*B. subtilis* ED24 was spotted onto a CMC agar plate [18] and incubated at 30 °C for 48 h. The plate was then thoroughly soaked with Congo red, followed by 1 M NaCl. The presence of halos indicated the production of extracellular cellulase.

#### ACC Deaminase Production

*B. subtilis ED24* was evaluated on Dworkin and Foster (DF) minimal sterile saline medium containing 3 mM ACC as the sole nitrogen source [19] and incubated at 30 °C for 3 days. ACC deaminase activity was determined by measuring α-ketobutyrate production at 540 nm.

#### Siderophore Production

*B. subtilis* ED24 was inoculated onto a universal Chrome Azurol S (CAS) agar plate (Sigma-Aldrich, Casablanca, Morocco) [20] and incubated at 30 °C for 7 days. The formation of an orange halo around the colony indicated siderophore production.

#### Extraction and Identification of Secondary Metabolites from B. subtilis ED24 and Fungal Co-cultures

Metabolites produced by *B. subtilis* ED24 were analyzed using gas chromatography-mass spectrometry (SCION 8900 Triple Quadrupole GC–MS). The bacterial strain was co-cultured with different fungi in PDB medium at 28 °C for 5 days. After incubation, the culture was filtered, and ethyl acetate (twice the volume of the filtrate) was added to extract metabolites. The mixture was shaken thoroughly, and the organic phase (upper) was collected. This extraction process was repeated three times, reusing the aqueous phase for each extraction. The organic extract was treated with anhydrous sodium sulfate (~ 10–15 g) to remove residual water, filtered, and evaporated using a rotary evaporator at 40 °C. The concentrated extract was redissolved in ethyl acetate. Aliquots (2 mL) of the solubilized metabolites were analyzed by GC–MS. The oven temperature program started at 50 °C, ramped to 300 °C at 5.5 °C/min, with a hold time of 2 min at the final temperature. The injection was conducted in standard mode, with a total runtime of 48.5 min. The analysis was performed in electron ionization (EI) mode, with a source temperature of 230 °C and electron energy of 70 eV. Detection was carried out in Q1MS mode over a mass range of 50–500 amu, with a scan time of 1000 ms. Data processing involved Gaussian smoothing, RT filtering, and a signal-to-noise ratio threshold of metabolites identified using the NIST Tandem Mass Spectral Library (NIST 20), achieving predictability greater than 90% [21].

Metabolites were detected using GC–MS and were annotated using the MS NIST 2014 library, ensuring a match probability exceeding 90%. Structural classification was performed using the PubChem and LIPID MAPS databases. To mitigate heteroscedasticity and pseudo-scaling effects, a power transformation was applied [22]. Metabolite quantification was derived from peak area integration, with dodecane serving as the internal standard. The Gene-to-KEGG Orthology (*G2KO*) tool developed by IIT Mandi [23] was used to assign KEGG codes to microbial strains obtained from metagenomic data. These codes were used in the KEFF Mapper tool to reconstruct metabolic pathways. The resulting pathway models were then graphically represented using BioRender. For data representation, a heatmap was generated in R to display compound abundance patterns, and an UpSet plot was created using the *UpSetR* package to illustrate shared and unique metabolites across different conditions*.*

### Effect of B. subtilis ED24 on Seeds Germination, Growth, and Yield Parameters of Durum Wheat Infected with F. culmorum

#### B. subtilis ED24 Biocontrol Activity Against F. culmorum in Wheat Under Greenhouse Conditions

To assess the biocontrol potential of *B. subtilis* ED24 against *F. culmorum* in wheat, a potted plant experiment was conducted at the Agriculture Innovation and Technology Transfer Centre (AITTC) of UM6P in Benguerir, Morocco, in December 2022 under natural daylight conditions. Wheat seeds (variety: Karim, *Triticum durum*, characterized by early maturity, moderate plant height, and good adaptability to semi-arid conditions [24]) were surface disinfected by soaking in 70% ethanol (Sigma-Aldrich, Casablanca, Morocco) for 2 min and thoroughly washed with sterile water. Seeds were then treated with a 1% sodium hypochlorite solution for 3 min, followed by another three rinses with sterile water, and subsequently dried under a laminar flow hood [25]. To confirm sterility, 100 µl of the final rinse water was plated on TSA and PDA and incubated at 30 °C for 24 h and 7 days, respectively; as a result, no microbial growth was observed. The experimental design consisted of three treatments, with the 1 st treatment representing the infected control group (C-) where seeds were infected with *F. culmorum* by soaking in a spore suspension for 30 min then dried under a sterile hood. The spore suspension was prepared by gently scraping a *F. culmorum* culture plate with a sterile spatula, filtering the suspension through muslin to separate spores from mycelium. The spore concentration was determined using a Neubauer counting chamber under a light microscope and adjusted to 10^6^ spores/mL by dilution with sterile distilled water. The 2nd treatment consisted of the biocontrol treatment (ED24) where seeds were first infected with *F. culmorum* as described above then immersed in a *B. subtilis* ED24 suspension for 30 min and subsequently dried under a sterile hood. The bacterial suspension was prepared by culturing *B. subtilis* ED24 in TSB at 30 °C for 24 h with shaking at 180 rpm. The culture was adjusted to a final concentration of 10^8^ CFU/mL, verified using the Quantom™ XT Microbial Cell Counter. The 3rd treatment corresponds to the chemical control group (TEBU), where seeds were first infected with *F. culmorum* and treated with a tebuconazole solution (600 mL/100 kg) for 10 min and then dried under a hood.

The treated seeds were sown in pots (12 seeds per pot of 4 kg) containing a plant growth substrate mixture of 25% sand, 25% perlite, and 50% agricultural soil collected from the AITTC at UM6P in Benguerir, central Morocco (32° 13′ 11.5″ N, 7° 53′ 29.9″ W). The sampled soil was characterized as phosphorus-deficient (Olsen P: 3 ppm), alkaline (pH 8.24), and low in organic matter (1.20%). At the full tillering stage, approximately 43 days after sowing, *B. subtilis* ED24 treated plants were inoculated with 20 mL of bacterial suspension (10^8^ CFU/mL) to ensure continued rhizosphere colonization and sustained biocontrol activity; in contrast, TEBU was applied once at the pre-sowing stage in accordance with the manufacturer’s recommendations and standard agronomic practices. This seed coating method ensures uniform application, minimizes stripping, and promotes strong adhesion to the seed surface [26].

Germination rate was assessed, considering damping-off symptoms as a key indicator of seedling establishment and early plant health. Growth and yield parameters were measured at the harvest stage, approximately 115 days after sowing [27]. Yield parameters, including thousand grain weight and number of grains per pot, were measured using a near-infrared spectrometer (NIRS). Morphometric parameters, including leaf area measured with a leaf area meter and root traits assessed using the WinRHIZO, were recorded from all germinated plants per pot. The values were averaged to obtain a composite measurement for each pot, and five replicate pots were used per treatment. In addition, biochemical parameters including shoot nutrient contents (NPK) were analyzed by inductively coupled plasma optical emission spectrometry (ICP-OES), and the biochemical composition of harvested seeds was analyzed using Fourier-transform infrared (FTIR) spectroscopy. Rhizospheric soil was sampled and immediately stored at − 20 °C for subsequent DNA extraction and metabarcoding analysis. The germination rate and seed vigor index were calculated using the following formulas:

- Germination rate (%) = (number of germinated seeds ÷ total number of seeds) × 100
- Seed vigor index (SVI) = germination percentage × seedling length (cm)

#### Isolation and Identification of F. culmorum Associated with Harvested Wheat Roots

To evaluate the biocontrol efficacy of *B. subtilis* ED24 against *F. culmorum*, the presence of *F. culmorum* spores in wheat roots was assessed to confirm the pathogen’s colonization under *B. subtilis* ED24, TEBU, and infected control (C-) groups. The root fragments (1 cm in length) were excised, washed with distilled water, and surface sterilized by soaking in 70% ethanol (v/v) for 5 min, followed by immersion in a 2% (v/v) sodium hypochlorite solution for 5 min. The fragments were then rinsed three times with sterile water and dried on sterile blotting paper before being placed in Petri dishes containing PDA supplemented with 100 µg/mL of chloramphenicol, a broad-spectrum antibiotic used to inhibit bacterial growth. The cultures were incubated at 27 °C for 7 days. Purification of fungal isolates was performed on PDA under the same culture conditions. For identification, hyphae were fixed in 2.5% glutaraldehyde in 0.1 M phosphate buffer (pH 7.2) and subsequently visualized using a light microscope and atomic force microscopy (AFM) (Anton Paar) [28].

#### DNA Extraction and High-Throughput Miseq Sequencing

Total DNA from the rhizospheric soil of harvested wheat plants was extracted using the QIAcube system following the manufacturer’s protocol (DNeasy Plant Pro Kit, QIAGEN). The purity and concentration of the extracted DNA were quantified using a Qubit 4 Fluorometer (ThermoFisher). Subsequently, PCR amplification was performed to target both fungal ITS2 and bacterial 16S rRNA genes. For bacterial community analysis, the V3-V4 region of the 16S rRNA gene was amplified using the primers 341 F (CCTAYGGGRBGCASCAG) and 806R (GGACTACNNGGGTATCTAAT). For fungal community analysis, the ITS2 region was amplified using the primers ITS1F (GCATCGATGAAGAACGCAGC) and ITS2R (TCCTCCGCTTATTGATATGC). The resulting libraries were prepared according to the Illumina metagenomic workflow and sequenced on the Illumina MiSeq platform. Reads of the sequence data have been deposited in the NCBI Sequence Read Archive (SRA) with accession number PRJNA1226402.

#### Bioinformatics Processing and Taxonomic Annotation

The raw FASTQ files were processed in R version 4.2.2 using the DADA2 pipeline [29] to ensure high-quality sequence analysis. Low-quality reads with an average Phred score below 30 were discarded to minimize sequencing errors. Bacterial sequences were filtered and trimmed to remove residual primer and adapter sequences, and error rate estimation was performed to refine the sequence inference process. Forward and reverse reads were merged using the *mergePairs* function in DADA2 to reconstruct high-resolution amplicon sequence variants (ASVs). Taxonomic classification of bacterial ASVs was conducted using the SILVA reference database [30], while fungal ASVs were assigned against the UNITE database [31].

### Statistical Analysis

The statistical significance of germination and growth data was determined using the statistical package for the social sciences software (GraphPad Prism 8). The comparison between treatments was performed using one-way analysis of variance (one-way ANOVA) followed by mean comparisons according to the post-hoc analysis with Tukey’s test. Significant differences were set at *p* < 0.05. Microbial beta diversity was analyzed with a Bray–Curtis dissimilarity matrix and visualized through non-metric multidimensional scaling (nMDS) to assess species composition variations in bacterial and fungal communities across the tested conditions. Differences between microbial community compositions were tested for significance using PERMANOVA (999 permutations) in PRIMER 7 (Primer-E Ltd, Plymouth, UK). Heatmaps, generated with the Complex Heatmap package in R (version 3.3.2), displayed the 100 most abundant fungal and bacterial taxa to highlight compositional variations at finer taxonomic levels. Multivariate analyses, including numerical clustering (PRIMER 7) and principal component analysis (PCA) using the “factoextra” package in R, were applied to data matrices from microbial taxa clusters and wheat growth parameters.

## Results

### Determination of Anti-fungal Activity of B. subtilis ED24

**Fig. 1:**
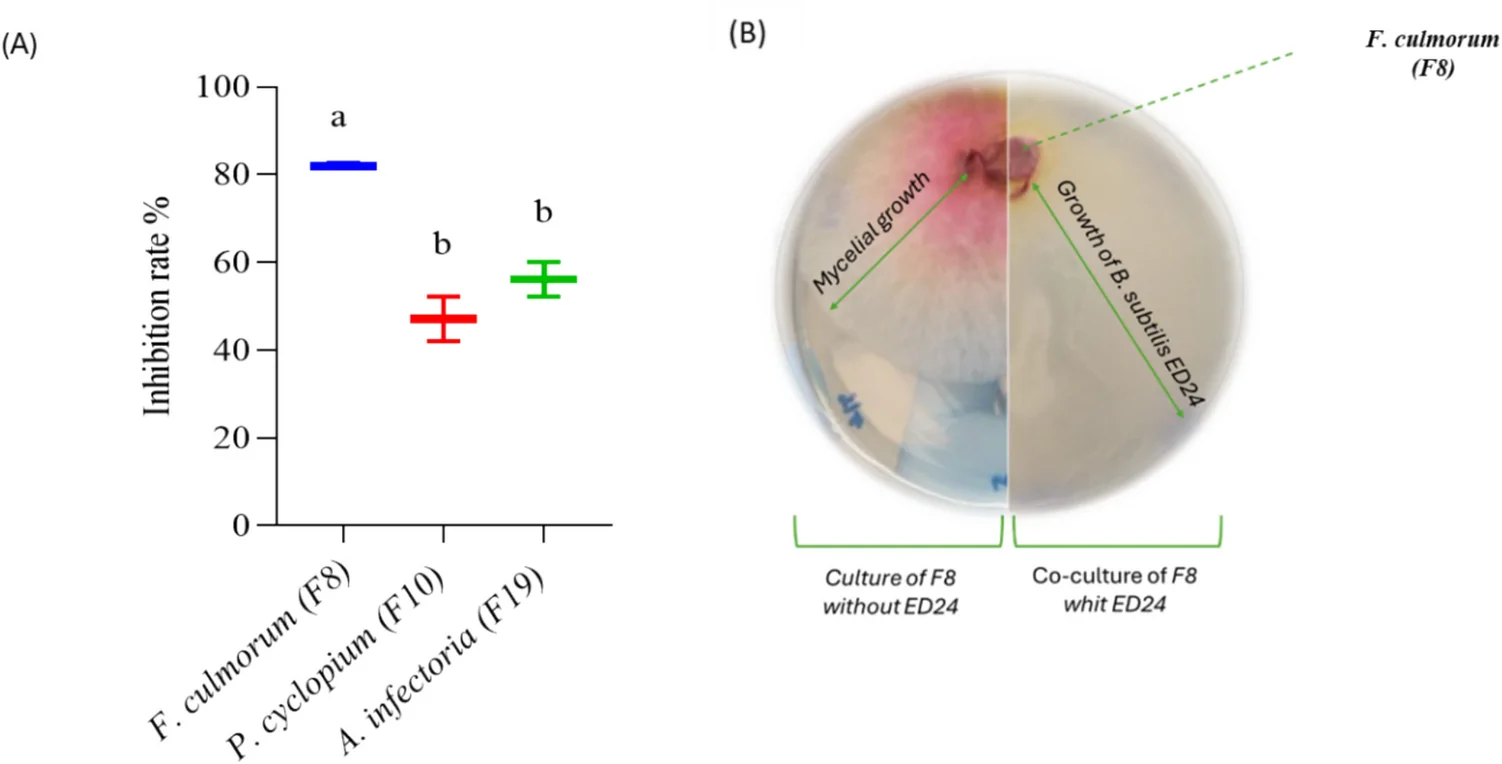
**A** Effect of *B. subtilis* ED24 on mycelial growth of the fungi after 5 days of incubation. Vertical bars represent means and standard errors (mean ± SE) based on two replicates. Distinct lowercase letters indicate statistical significance at *p* ≤ 0.05 between each treatment and its control. **B** Effect of *B. subtilis* ED24 on *F. culmorum* growth after 5 days of incubation

In vitro assays revealed that *B. subtilis* ED24 effectively suppressed fungal growth (Fig. 1), resulting in a significant reduction in mycelial surface area across all tested fungi. Co-inoculation with *B. subtilis* ED24 led to significant inhibition rates of 82% for *F. culmorum*, 56% for *A. infectoria* (F19), and 47% for *P. cyclopium* (F10).

### Biocontrol Related Traits and Metabolomic Profiling of B. subtilis ED24

Enzymatic assays revealed that *B. subtilis* ED24 possesses multiple biocontrol traits. It exhibited notable cellulolytic activity, forming a 5.5 mm clearance zone. *B. subtilis* ED24 also showed ACC deaminase activity, reaching 0.32 nmol per mL per hour. However, no proteolytic activity was detected. In addition, *B. subtilis* ED24 demonstrated the ability to produce siderophores, as indicated by a 2.7 mm halo.

**Fig. 2:**
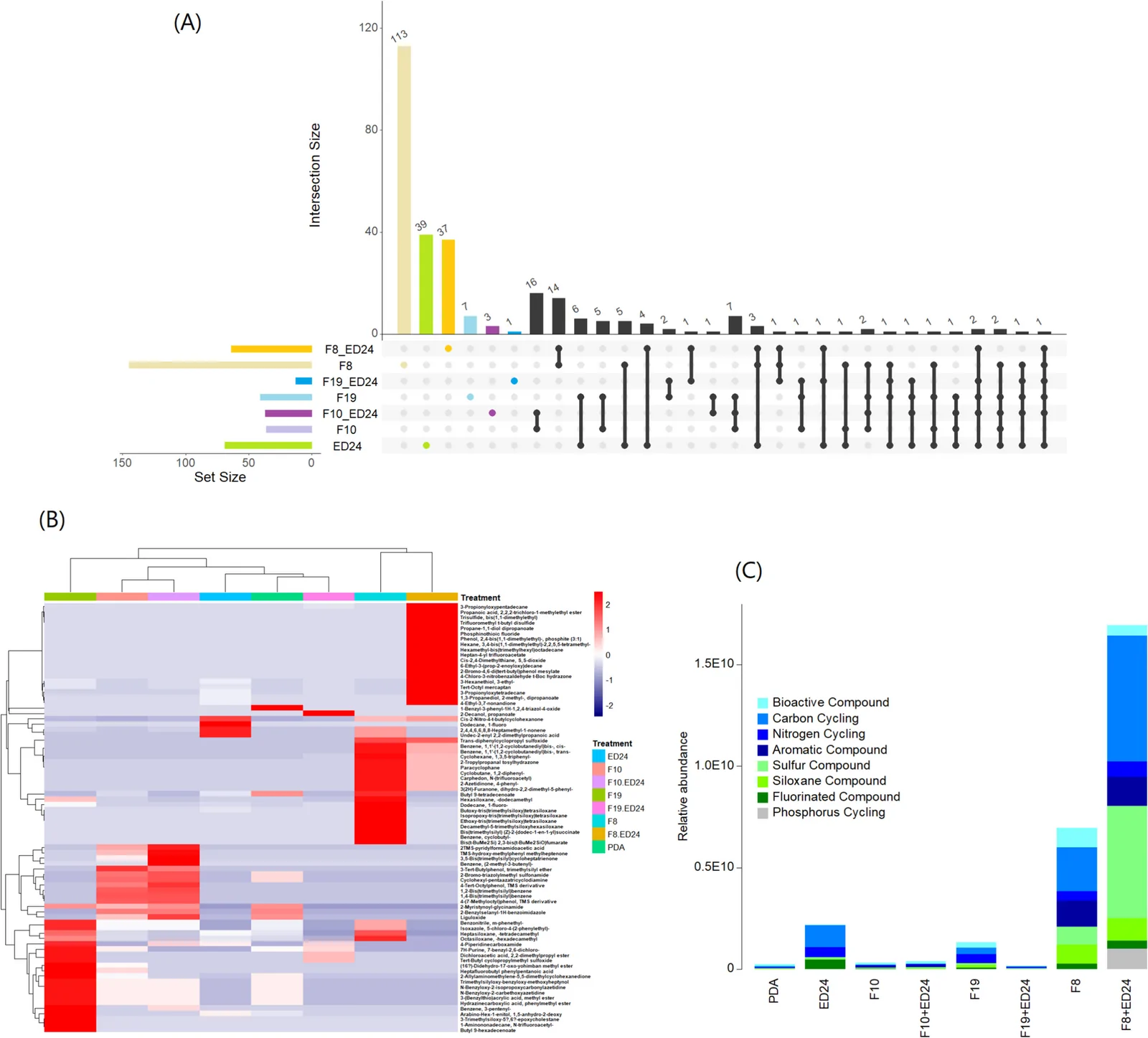
Comparative visualization of microbial metabolite profiles. **A** Intersection analysis of shared and unique metabolites across treatments, **B** heatmap of relative abundances of metabolites across treatments, and **C** relative abundance of compounds across treatments

The metabolomic analysis revealed that under individual culture conditions, *F. culmorum* (F8) exhibited the highest number of specific metabolites (113), followed by *B. subtilis* ED24 with 39, *A. infectoria* (F19) with 7, and *P. cyclopium* (F10) which produced none. However, co-inoculation of *B. subtilis* ED24 with *F. culmorum* (F8) resulted in the production of 37 exclusive metabolites, while co-inoculation with *P. cyclopium* (F10) and *A. infectoria* (F19) led to the detection of 3 and 1 unique metabolites, respectively (Fig. 2A).

Heatmap and hierarchical cluster analyses revealed distinct metabolomic profiles across treatments. The co-inoculation of *B. subtilis* ED24 with *F. culmorum* (F8) exhibited the most pronounced metabolic activity, a pattern that was not observed in single-strain treatments. Similarly, the co-inoculation of *B. subtilis* ED24 with *A. infectoria* (F19) induced specific metabolite production. In contrast, the co-inoculation of *B. subtilis* ED24 with *P. cyclopium* (F10) showed only minor metabolic changes (Fig. 2B).

Notably, co-inoculation of *B. subtilis* ED24 with *F. culmorum* (F8) led to a higher production of carbon cycling compounds, phosphorus-cycling compounds, and sulfur compounds—metabolites that were absent in single-strain treatments. Additionally, co-inoculation with *P. cyclopium* (F10) resulted in a slight overproduction of sulfur compounds. In contrast, co-inoculation with *A. infectoria* (F19) significantly reduced nitrogen cycling, carbon cycling, and suppressed fluorinated, siloxane, aromatic, and bioactive compounds (Fig. 2C).

### In Planta ED24-Fungus Inoculation Experiment

#### Effect of B. subtilis ED24 on Seed Germination Parameters

**Fig. 3:**
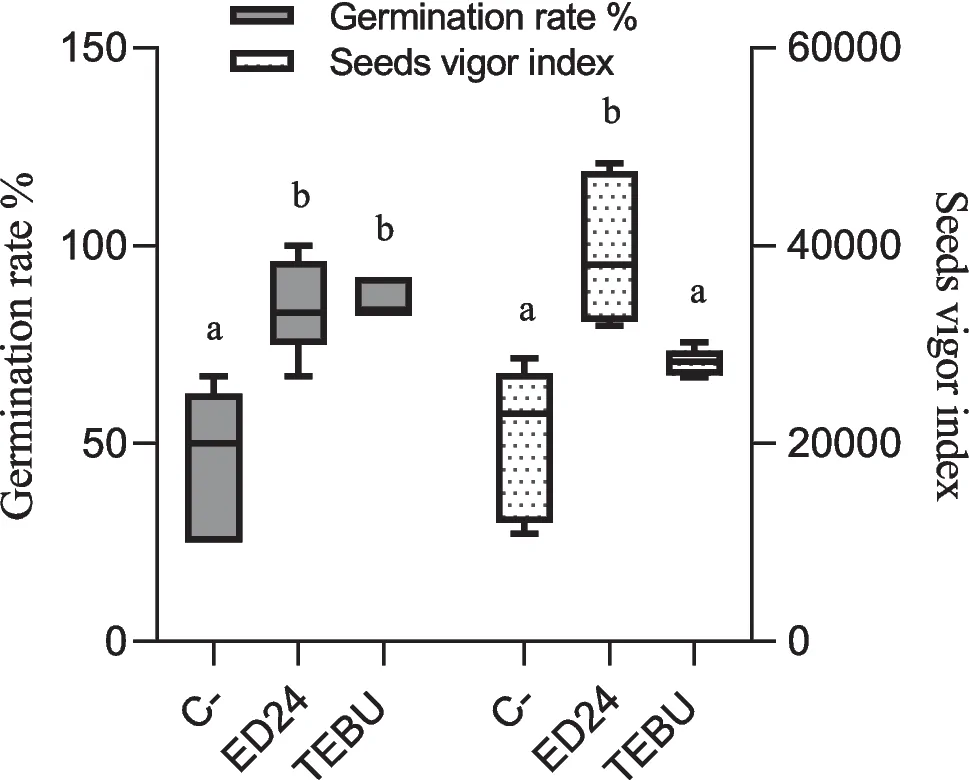
Effect of *B. subtilis* ED24 and TEBU treatments on wheat seed germination rate measured 7 days after sowing and their vigor index. Vertical bars depict the means and standard errors (mean ± SE) based on five replicates. Distinct lowercase letters indicate statistical significance at *p* ≤ 0.05

Seeds infected with *F. culmorum* (F8) and subsequently treated with *B. subtilis* ED24 or TEBU exhibited a significantly higher germination rate compared to the infected control group (C-) (Fig. 3). Specifically, seeds treated with *B. subtilis* ED24 and TEBU exhibited germination rates of 85% and 86.6%, respectively, whereas the infected control group (C-) showed only 45% germination. Moreover, seed vigor index values revealed that seeds treated with *B. subtilis* ED24 demonstrated significantly higher vigor, while seeds of the infected control group (C-) and TEBU treatment exhibited lower vigor index*.*

#### Effect of B. subtilis ED24 on Wheat Plant Growth Parameters

##### Effect on Wheat Plant Morphological Parameters

 Analysis of morphological growth parameters (Table 1) revealed that *B. subtilis* ED24 treatment significantly enhanced leaf area (47.75 cm^2^), stem height (46.46 cm), spike length (6.96 cm), and shoot dry weight (3.96 g) compared to TEBU treatment (21.66 cm^2^, 32.6 cm, 5.16 cm, and 1.81 g), respectively. However, no significant differences were observed between *B. subtilis* ED24 and the infected control (C-) groups in terms of stem height and spike length, with infected control group (C-) values of 44.92 cm^2^, 45.86 cm, and 8.09 cm, respectively.

**Table 1:** Stem height, spike length, leaf area, and shoots dry weight of wheat plants under different treatments. Data are means of five replicates, and each replicate consists of five wheat plants per pot. Distinct lowercase letters indicate statistical significance at *p* ≤ 0.05

Treatments	Morphological parameters				
	**Stem height (cm)**	**Leaf area (cm**^**2**^**)**	**Spike length (cm)**		**Shoots dry weight (g)**
**C-**	45.86^b^	44.92^b^	8.09^b^	3.45^b^	
**ED24**	46.46^b^	47.75^b^	6.96^b^	3.96^b^	
**TEBU**	32.6^a^	21.66^a^	5.16^a^	1.81^a^	

##### Effect on Shoot N, P, K Contents

**Fig. 4:**
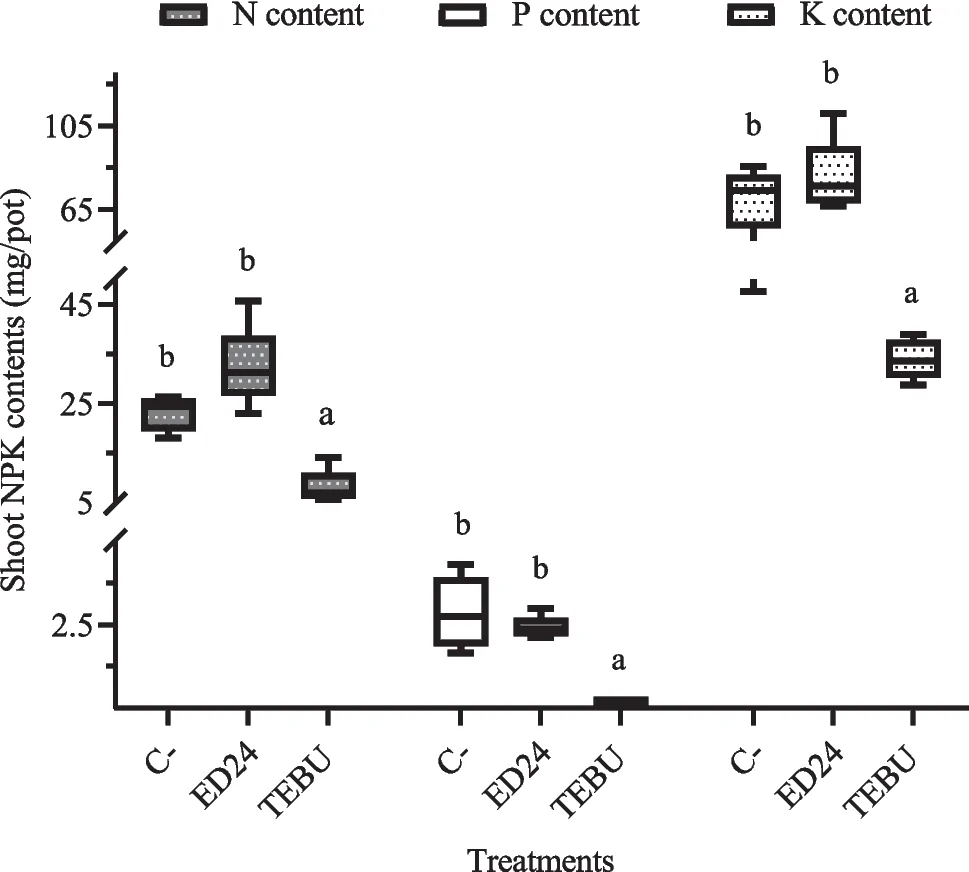
Effect of *B. subtilis* ED24 and TEBU treatments on the shoot nitrogen, phosphorus, and potassium contents. Vertical bars depict the means and standard errors (Mean ± SE) based on five replicates, and each replicate consists of five wheat plants per pot. Distinct lowercase letters indicate statistical significance at *p* ≤ 0.05

Shoot nutrient analysis revealed significant differences in N, P, and K contents among treatments (Fig. 4). Plants treated with *B. subtilis* ED24 demonstrated significantly higher N (32.34 mg/pot), P (2.4 mg/pot), and K (80.56 mg/pot) levels, compared to those treated with TEBU, accumulating 8.11, 0.18, and 33.98 mg/pot (N, P, K), respectively. However, no significant differences were observed between *B. subtilis* ED24 treatment and the infected control group (C-) in terms of N (23.07 mg/pot), P (2.86 mg/pot), and K contents (69.87 mg/pot).

##### Effect on Wheat Yield and Seed Biochemical Quality

Analysis of wheat yield parameters revealed that *B. subtilis* ED24 treatment significantly increased the thousand grain weight to 40.42 g compared to 29.1 g under TEBU treatment (Fig. S.1). However, no significant difference was observed in the number of grains per pot between the two treatments, with *B. subtilis* ED24 and TEBU treatments yielding 113 and 104 grains, respectively. Biochemical analysis of harvested grains showed that *B. subtilis* ED24 treatment significantly enhanced seed protein and ash content, reaching 19.98% and 1.95%, respectively. These values were higher compared to both TEBU treated and infected control group (C-) plants, which exhibited protein levels of 19.09% and 18.31% and ash content of 1.91% and 1.74%, respectively. Regarding seed fat and moisture content, no significant differences were observed among treatments. However, TEBU treatment led to increased crude fiber (2.56%) and starch content (51.96%) compared to the infected control group (C-) (2.53%, 48.84%) and *B. subtilis* ED24 treatment (2.74%, 46.81%) (Table 2).

**Table 2:** Number of seeds per pot, 1000 seed weight (TGW), proteins, fat, crude fiber, starch, ASH, moisture of harvested wheat seeds of different treatments. Data are based on five replicates, and each replicate consists of five wheat plants per pot. Distinct lowercase letters indicate statistical significance at *p* ≤ 0.05

Treatments	Number of seeds/pots	TGW (g)	Proteins %	Fat %	Crude fiber %	Starch %	ASH %	Moisture %
C-	57.2^a^	43.27^b^	19.09^a^	1.23^a^	2.53^ab^	48.84^ab^	1.91^b^	9.37^a^
ED24	113.8^b^	40.44^b^	19.98^b^	1.28^a^	2.47^a^	46.81^a^	1.95^b^	9.49^a^
TEBU	104.2^b^	29.1^a^	18.31^a^	1.2^a^	2.56^b^	51.96^b^	1.74^a^	9.19^a^

#### Effect on Wheat Roots Morphology and F. culmorum Spore Colonization

**Fig. 5:**
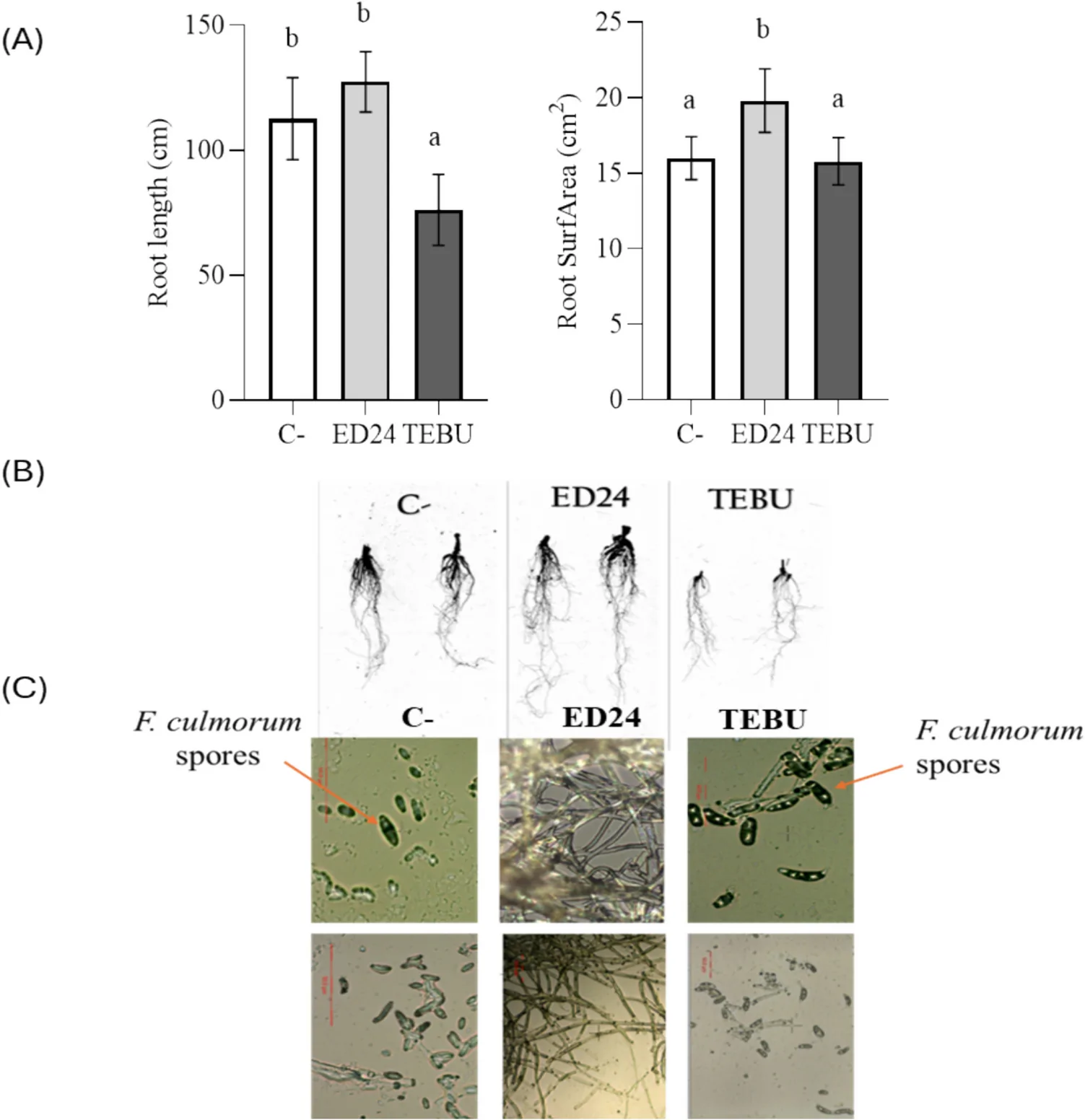
Effect of *B. subtilis* ED24 and TEBU treatments on wheat root morphology and *F. culmorum* spore colonization. **A** Wheat root length and surface area under *B. subtilis* ED24 and TEBU treatments. **B** Root system architecture of wheat plants treated with *B. subtilis* ED24 and TEBU, scanned, and analyzed using WinRHIZO. **C** Microscopic observations of *F. culmorum* spores under *B. subtilis* ED24 and TEBU treatments; the presence of spores is highlighted with orange arrows

Analysis of root morphology demonstrated that plants treated with *B. subtilis* ED24 exhibited significantly higher root length (127.4 cm) compared to TEBU treatment (76.1 cm). However, no significant difference was observed between the *B. subtilis* ED24 and the infected control group (C-) (112.6 cm) (Fig. 5A). Additionally, *B. subtilis* ED24 significantly increased surface area (19.8 cm^2^) compared to both TEBU and infected control (C-) groups, with values of 15.8 cm^2^ and 16 cm^2^, respectively (Fig. 5B). The microscopic visualization of endophytic fungi showed the abundance of *F. culmorum* spores in the root endosphere of both the infected control group (C-) and TEBU treatments. Conversely, *F. culmorum* spores were notably absent in the root endosphere of wheat plants treated with *B. subtilis* ED24 (Fig. 5C).

#### Effect of B. subtilis ED24 on the Abundance and Diversity of Microorganisms in the Wheat Root Rhizosphere

**Fig. 6:**
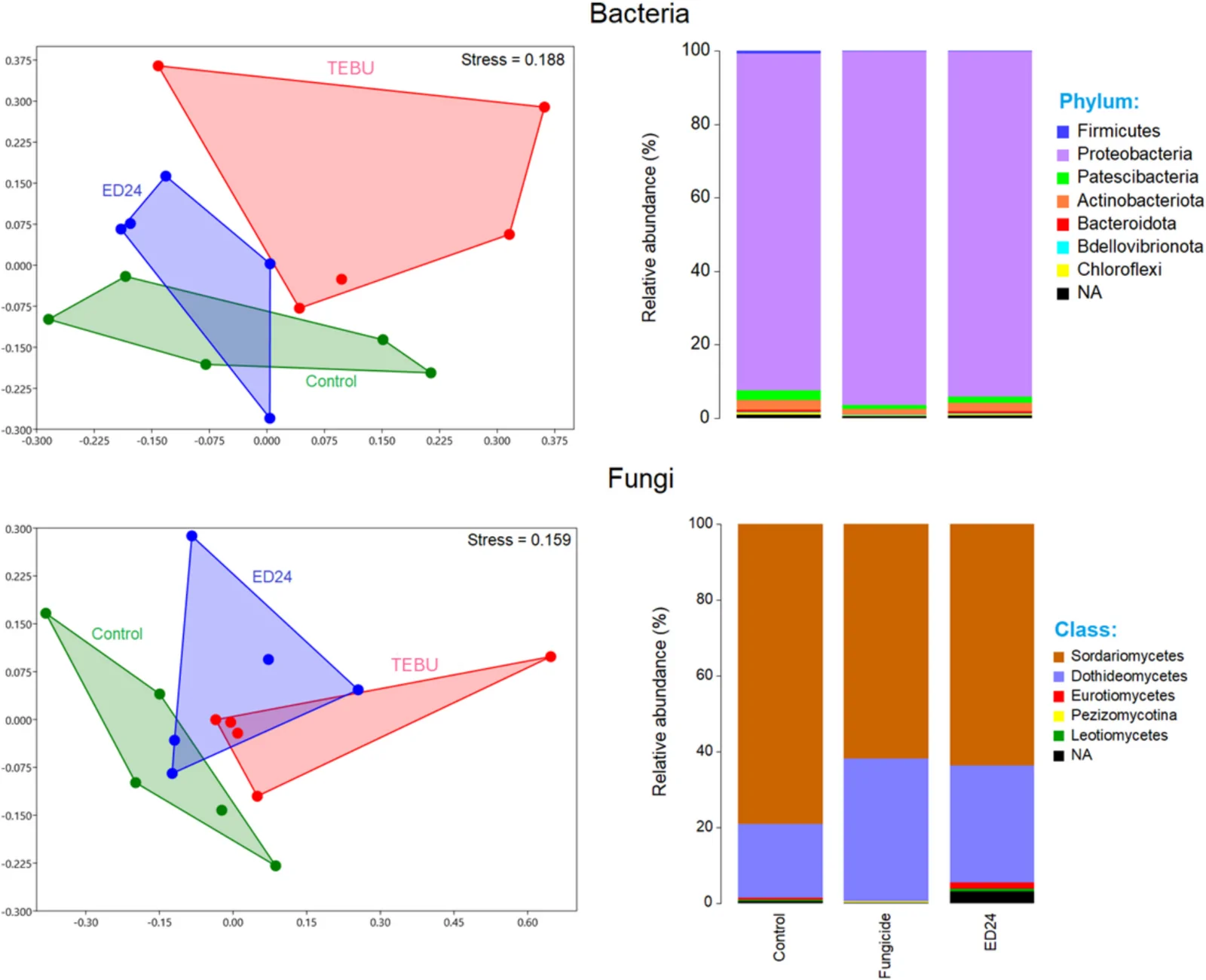
Relative abundance and NMDS analysis of wheat rhizospheric bacteria and fungi communities in infected control group (C-), *B. subtilis* ED24, and TEBU treatments

At the class level, fungal community analysis showed that both *B. subtilis* ED24 and the TEBU treatments reduced the abundance of *Sordariomycetes*, although this class remained dominant across all treatments. Conversely, both treatments led to an increase in *Dothideomycetes*. Notably, *B. subtilis* ED24 treatment also increased the abundance of *Eurotiomycetes* compared to the other treatments (Fig. 6). For bacterial communities, Proteobacteria was the most dominant phylum across all treatments. However, the TEBU treatment resulted in a decrease in Actinobacteria and Patescibacteria. Non-metric dimensional scaling (NMDS) analysis revealed clear clustering patterns, indicating distinct microbial community structures under each treatment for both bacteria and fungi.

**Fig. 7:**
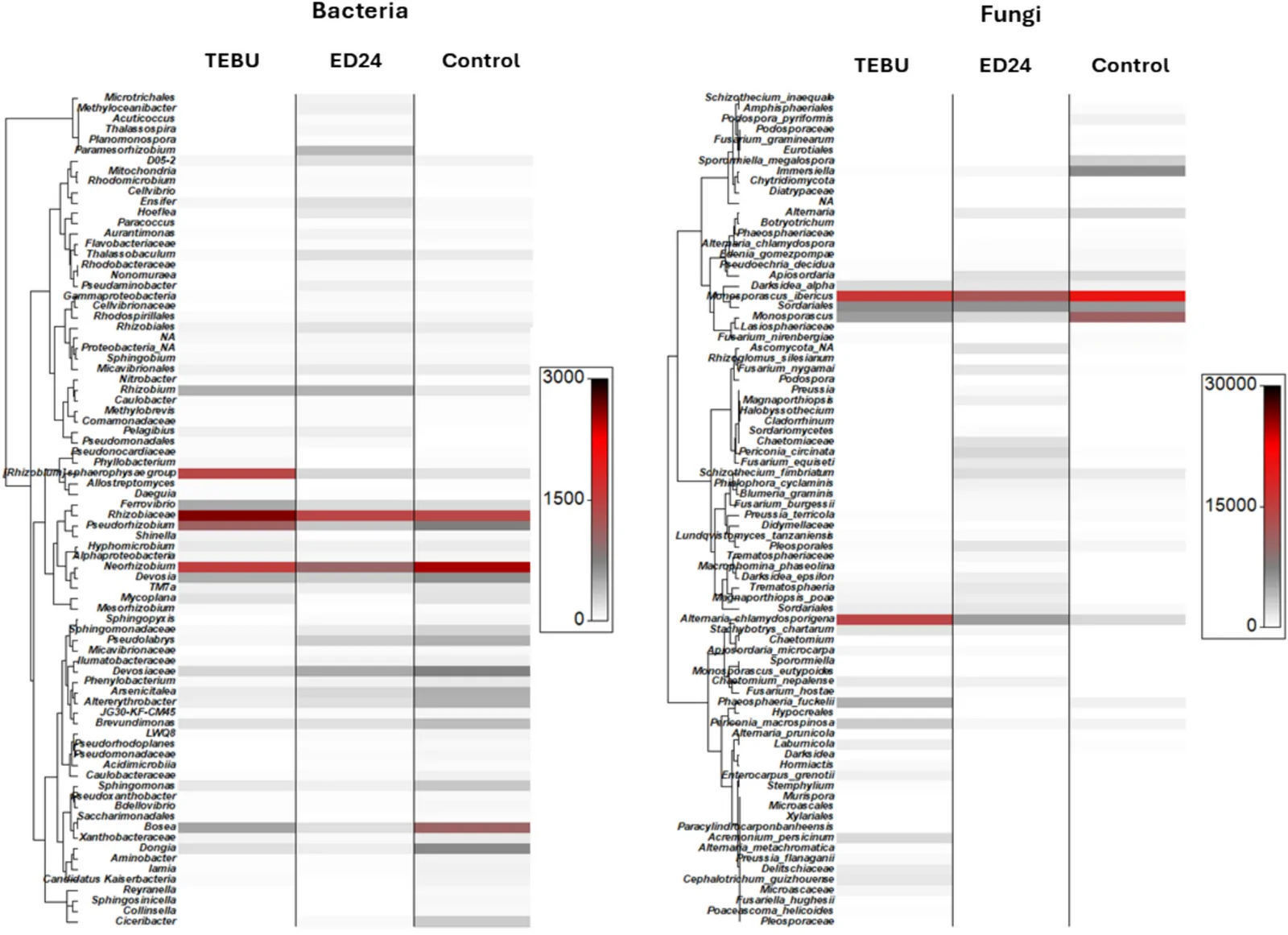
Heatmap displays the relative abundance of the 100 most frequent amplicon sequence variants in the bacterial and fungal communities of the wheat rhizosphere of the infected control group (C-), *B. subtilis* ED24, and TEBU treatments

The heatmap analysis of the 100 most abundant taxa revealed distinct microbial community shifts across treatments (Fig. 7). For the bacterial community, *B. subtilis* ED24 treatment led to an increased abundance of *Paramesorhizobium*, *Ensifer*, *Hoeflea*, and Rhizobiales*.* In contrast, TEBU treatment resulted in a higher abundance of the *Sphaerophysae* group, *Pseudorhizobium*, and Rhizobiaceae. The infected control group (C-) exhibited a greater abundance of *Neorhizobium*, Devociaceae, *Bosea*, *Dongia*, *Sphingomonas*, and *Brevundimonas*. For the fungal community, TEBU fungicide treatment promoted the abundance of *Alternaria chlamydosporigena*, *Phaeosphaeria fuckelii*, *Periconia macrospinosa*, *Laburnicola*, and *Acremonium persicinum*. In contrast, *B. subtilis* ED24 favored a higher abundance of *Rhizoglomus silesianum*, *Magnaporthiopsis*, *Periconia circinata*, and Pleosporales. The infected control group (C-) was dominated by *Immersiella*, *Sporormiella megalospora*, *Alternaria*, and *Monosporascus*.

**Fig. 8:**
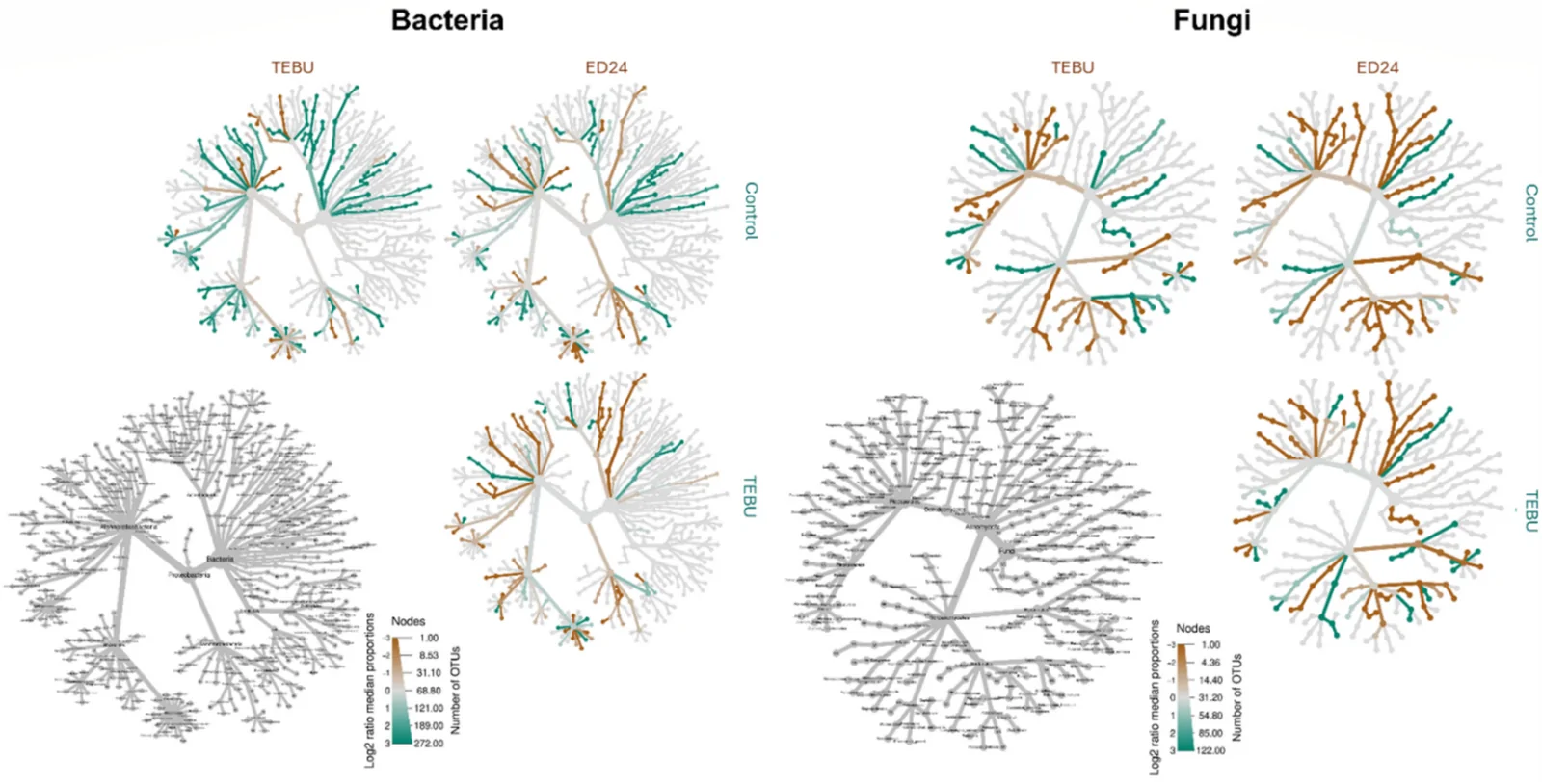
Heat stress illustrating the wheat rhizospheric bacteria and fungi distribution under *B. subtilis* ED24 and TEBU treatments compared to the infected control group (C-)

The differential heat tree analysis reveals significant shifts in bacterial and fungal community composition under the *B. subtilis* ED24 treatment compared to infected control group (C-) and TEBU treatment (Fig. 8). In the bacterial community, taxa from the Alphaproteobacteria and Actinobacteria phyla appear more enriched in *B. subtilis* ED24 than in infected control group (C-) and TEBU groups. Specifically, certain Rhizobiales members show increased abundance. In contrast, some Proteobacteria taxa, which are dominant in the infected control group (C-), show a reduction in *B. subtilis* ED24. Similarly, in the fungal community, members of the Ascomycota phylum, particularly taxa related to *Dothideomycetes*, exhibit enrichment in *B. subtilis* ED24 compared to the other treatments. Conversely, some fungal taxa that are abundant under the infected control group (C-), such as *Basidiomycota* members, are suppressed in *B. subtilis* ED24.

**Fig. 9:**
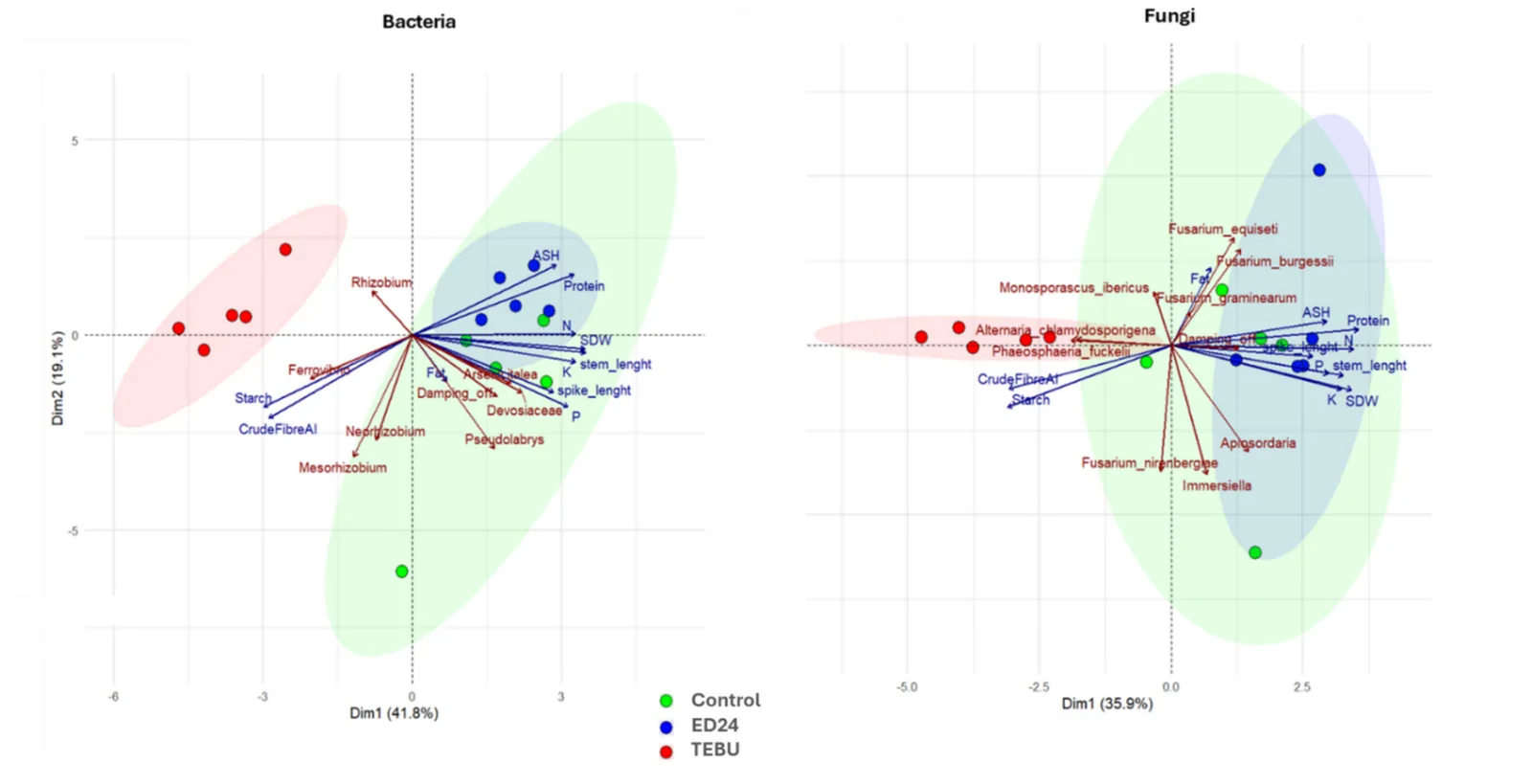
Principal component analysis (PCA) illustrating wheat rhizospheric bacteria and fungi distribution along with plant growth parameters under *B. subtilis* ED24 and TEBU treatments

The PCA of bacterial taxa explains 59.66% of the total variance, with Factor 1 accounting for 32.55% and Factor 2 explaining 27.11% of the variability (Fig. 9, left panel). The distribution of bacterial taxa and plant-related variables highlights distinct microbial associations with plant health and soil fertility. Several nitrogen-fixing bacterial genera, including *Mesorhizobium*, *Neorhizobium*, *Rhizobium*, and *Ferrovirbio*, are positioned in the negative region of Factor 1. These taxa show negative correlations with plant growth parameters such as stem height, SDW, and P. Conversely, *Arsenicitalea* and Devosiaceae are positively associated with P availability and damping-off disease incidence. On the other hand, the PCA for the fungal community accounts for 55.99% of the total variance, with Factor 1 contributing 29.80% and Factor 2 explaining 26.19%. The distribution of fungal taxa suggests strong associations between fungal diversity and plant growth traits (Fig. 9, right panel). A striking feature of the fungal PCA is the clustering of *Fusarium* species, including *Fusarium graminearum*, *Fusarium equiseti*, and *Fusarium *spp., which are strongly associated with damping-off disease. In contrast, taxa such as *Alternaria chlamydosporigena* and *Phaeosphaeria fuckelii* are negatively correlated with plant health parameters.

## Discussion

*Bacillus *spp. have been frequently recognized for their broad spectrum of antifungal properties [32, 33]. In this study, the antifungal activity of *B. subtilis* ED24 against phytopathogenic fungi is associated with the presence of novel metabolites that were not found in single-strain treatments (Fig. 2). These include carbon cycling compounds, such as propionic acid (PPA) and its precursors, propanediol, and propionyloxytetradecane, known to induce apoptotic fungal cell death through reactive oxygen species (ROS) accumulation, metacaspase activation, and cell cycle arrest. Alongside carbon cycling compounds, phosphorus-cycling compounds like phosphite and organic phosphorus derivatives are known to disrupt fungal metabolism by promoting polyphosphate and pyrophosphate accumulation and reducing ATP and NAD synthesis, ultimately impairing fungal growth [34–36]. While the antifungal mechanisms of these identified compounds are supported by literature, their specific contribution to the biocontrol activity of *B. subtilis* ED24 remains hypothetical, needing to be experimentally evidenced. Although several *Bacillus* species are known to inhibit fungal growth through the production of lipopeptides such as iturins, fengycins, and surfactins [37], the presence of lipopeptide biosynthetic genes in *B. subtilis* ED24 was not assessed in the current study, knowing that these compounds are well-documented antifungal agents. However, our findings can suggest additional research focusing on screening for these genes and quantifying lipopeptide production, as well as testing the activity of the detected metabolites individually to better elucidate the mechanisms underlying the observed antifungal activity.

Alongside the metabolomic profiling, the enzymatic activity of *B. subtilis* ED24 likely contributes significantly to its biocontrol potential. The production of cellulase may inhibit fungal development by degrading components of fungal cell walls, which include cellulose, chitin, glucan, and proteins [38]. In addition, *B. subtilis* ED24 demonstrated ACC deaminase activity, an important trait for enhancing plant tolerance to fungal pathogens [39]. This enzyme catalyzes the conversion of ACC (1-aminocyclopropane-1-carboxylate), the precursor of ethylene, into α-ketobutyrate and ammonia, thereby lowering ethylene levels, which is a stress-related plant hormone that tends to accumulate during biotic stress, inhibiting root growth and compromising plant defenses [40]. This reduction supports sustained root growth and facilitates the activation of plant defense pathways, contributing to improved resilience under fungal infection. Complementing enzymatic actions, the significant siderophore production is likely contributing to biocontrol by limiting iron availability, being an essential nutrient for fungal growth and vital biological processes, finding that corroborates with previous studies correlating *B. subtilis* reducing disease incidence (such as wilt caused by *Fusarium* spp.) with siderophore production [38] due to restricting iron availability and inhibition of pathogen proliferation [41].

The fungus *F. culmorum* is a major wheat pathogen, causing both pre- and post-emergence seedling death due to restricted water and nutrient movement [42, 43]. In this study, *F. culmorum* reduced germination and reduced seedling vigor; however, *B. subtilis* ED24 application (and TEBU as well) significantly enhanced germination (Fig. 3). It is well known that during the early stages of plant development, including germination and seedling growth, rhizospheric bacteria play a key role in enhancing the plant’s resistance to soil-borne pathogens [44, 45], but the extent of such a resistance remains instable as compared to chemical treatments. For instance, triazole group fungicides increase the germination rate [46]. The application of fungicides to seeds can effectively protect them from soil-borne infections by moving to the cotyledon, embryo, rootlet, and endosperm, utilizing the humidity in the soil to create a protective barrier against infections from the germination stage [46], while *B. subtilis* ED24 promoted germination by inhibiting *F. culmorum* pathogen growth, which helps reduce infection severity and improve seedling emergence [47]. Despite its protective effect on seed germination, TEBU significantly reduced wheat plant growth and yield compared to *B. subtilis* ED24 treatment and infected control group (C-) (Tables 1 and 2). Plants treated with TEBU exhibited reduced leaf area, stem height, spike length, root length, root surface area, shoot dry weight, and TGW of harvested wheat seeds. Previous studies have shown that triazole fungicides like TEBU suppress root development and negatively affect water metabolism and photosynthesis [48]. However, the infected control (C-) wheat plants that survived *F. culmorum* infection exhibited growth metrics comparable to those treated with *B. subtilis* ED24. This suggests that these seedlings may activate immune responses, potentially upregulating pathogen-response genes to combat the infection [27]. Research demonstrates that tolerance mechanisms in wheat involve genetic resistance, which initiates complex biochemical responses and metabolic shifts to counteract pathogen stress [49, 50]. The comparable growth performance between infected control group (C-) and *B. subtilis* ED24 treatment implies that *B. subtilis* ED24 primarily supports germination by mitigating the pathogen’s impact during early stages rather than directly influencing post-germination growth. *B. subtilis* ED24 not only uses its own metabolites to promote wheat growth and inhibit fungal pathogens but may also influence the surrounding rhizosphere microbes by encouraging beneficial microorganisms that support plant growth or by attracting other microbes that can help suppress the pathogen. In this study, treatment with *B. subtilis* ED24 significantly altered these communities, causing distinct shifts in fungal and bacterial populations (Fig. 6). Fungal community analysis at the class level showed that both *B. subtilis* ED24 and TEBU treatments led to a reduction in *Sordariomycetes* abundance, though this class remained dominant. *Sordariomycetes* includes various pathogenic and saprophytic fungi, and its suppression by *B. subtilis* ED24 suggests a competitive displacement of harmful taxa. In contrast, *B. subtilis* ED24 treatment increased the abundance of *Eurotiomycetes*, a class known for its association with plant-beneficial fungi, particularly those involved in stress tolerance and nutrient solubilization [51]. Additionally, an increase in *Dothideomycetes* under *B. subtilis* ED24 treatment suggests a potential restructuring of root fungal associations in favor of beneficial symbionts. Furthermore, the differential heat tree analysis revealed that *B. subtilis* ED24 favored the enrichment of fungal taxa associated with nutrient acquisition and plant growth, such as *Rhizoglomus silesianum* (Fig. 8). These fungi have been linked to mycorrhizal-like functions, enhancing phosphorus uptake and providing biotic stress tolerance [52, 53]. Conversely, TEBU treatment promoted the proliferation of fungal taxa such as *Alternaria chlamydosporigena* and *Phaeosphaeria fuckelii*, which have been associated with pathogenicity and reduced plant health [54, 55]. The negative correlation of these fungal taxa with wheat growth parameters indicates a potential trade-off between pathogen suppression and non-target effects of chemical fungicides (Fig. 9). Alongside the fungal community, analysis of the bacterial community demonstrated that *B. subtilis* ED24 induced a significant shift in root-associated microbiota, particularly enhancing beneficial taxa. Notably, *Paramesorhizobium*, *Ensifer*, and *Hoeflea* were enriched under *B. subtilis* ED24 treatment, all of which are known for their roles in nitrogen fixation, phytohormone production, and plant–microbe interactions [56, 57]. These bacteria contribute to improved nutrient availability and overall plant health [58]. In contrast, TEBU treatment resulted in an increased abundance of *Sphaerophysae*, *Pseudorhizobium*, and *Rhizobiaceae*, with some members negatively correlated with plant growth parameters. While *Rhizobiaceae* is typically associated with beneficial plant–microbe interactions, its specific members enriched by TEBU may reflect a shift toward opportunistic or less effective symbionts under chemical stress [59]. Additionally, TEBU significantly suppressed *Actinobacteria* and *Patescibacteria*, both of which play essential roles in organic matter decomposition, pathogen suppression, and plant resilience [60]. The PCA confirmed that *B. subtilis* ED24 treatment induced clustering of beneficial bacterial taxa, suggesting a restructured root microbiome more conducive to plant health. While nitrogen-fixing bacteria are generally regarded as beneficial, this study showed a negative correlation between their abundance and plant growth parameters. This counterintuitive pattern may be attributed to the stronger growth-promoting effects of other microbial taxa, which may have masked or outweighed the benefits typically associated with the N2 fixing bacteria such as *Neorhizobium*, *Rhizobium*. Moreover, unlike legumes, wheat does not perform symbiotic N2 fixation but can benefit from rhizobia as PGPR, whose PGP effects were not observed under our experimental conditions, likely due to the susceptibility of these bacterial groups to the pathogenic fungi. Additionally, *B. subtilis* ED24 treatment led to a notable suppression of *Mycoplasma* and mycoplasma-like organisms (MLOs), which have been linked to yield-reducing diseases in multiple crops [61, 62]. The reduction of MLOs under *B. subtilis* ED24 treatment further underscores its biocontrol potential in modulating disease-associated microbial taxa. The contrasting effects of *B. subtilis* ED24 and TEBU on root microbial communities highlight the potential risks of chemical fungicide use versus the benefits of biological control agents. TEBU treatment significantly altered fungal and bacterial compositions, leading to the proliferation of some pathogenic fungi and a decline in beneficial bacterial groups. In contrast, *B. subtilis* ED24 treatment fostered a microbial environment rich in plant growth-promoting and biocontrol-associated taxa, which may contribute to enhanced plant resilience against pathogens.

Overall, both *B. subtilis* ED24 and TEBU treatments significantly enhanced wheat germination rates, indicating their initial effectiveness in controlling early fungal infections. However, while TEBU treatment achieved fungal suppression, it also led to a significant reduction in final wheat yield, likely due to its broader toxicity affecting plant physiology or non-target beneficial microbes. In contrast, *B. subtilis* ED24 not only improved germination but also maintained healthy plant growth and yield, suggesting that its biocontrol effect is achieved without compromising plant performance. These findings underscore the potential of *B. subtilis* ED24 as a sustainable and environmentally friendly alternative to chemical fungicides in wheat protection strategies.

## Conclusion

The present study unlocked both the biocontrol and PGP properties of *B. subtilis* ED24, thus demonstrating a significant ecological contribution to control *F. culmorum*. Beyond its direct ability to suppress the pathogen, *B. subtilis* ED24 influences the root rhizosphere microbial community, fostering beneficial microbial interactions that boost plant resilience to biotic stress. This capacity to modulate rhizosphere-associated microbiomes represents a key mechanism for improving soil health and long-term agricultural sustainability. Overall, by reducing dependence on synthetic fungicides, the application of *B. subtilis* ED24 could contribute to environmental health and sustainability, supporting the transition toward more sustainable farming systems and global sustainable development goals. To further demonstrate this potential feature, research on molecular interactions with plant hosts and soil microbiota will help optimize its use in integrated disease management strategies. Additionally, field trials are crucial to validate its effectiveness in diverse environmental conditions and to facilitate its large-scale implementation as a reliable and environmentally sound biocontrol solution.

## Supplementary Information

Below is the link to the electronic supplementary material.

MOESM 1 (ODT 2,704 KB)

MOESM 2 (ODT 272 KB)

## Data Availability

Sequence data that support the findings of this study have been deposited in Sequence Read Archive with accession number PRJNA1226402.
