# Supplementary material for: Bacillus subtilis ED24 Controls Fusarium culmorum in Wheat Through Bioactive Metabolite Secretion and Modulation of Rhizosphere Microbiome
Source: Microb Ecol. 2025 Aug 19;88:89. doi: 10.1007/s00248-025-02590-5 (PMC12364970; doi:10.1007/s00248-025-02590-5)
Supplement: Supplementary file 1 [file 248_2025_2590_MOESM1_ESM.odt]

 
Figure 1: Propanoate metabolic pathway in B. subtilis ED24. 
Figure 1: Propanoate metabolic pathway in B. subtilis ED24. 

TEBUED24 
TEBU
ED24 
Figure 2: The effect of B. subtilis ED24 and tebuconazole treatments on spike growth in wheat.
